# Chronic Diffuse Nephritis

**Published:** 1887-01

**Authors:** A. L. Gihon

**Affiliations:** Medical-Director, United States Navy


### REPORT OF A CASE OF CHRONIC DIFFUSE NEPHRITIS, AND THE EFFICACY OF BASHAM'S MIXTURE.

*By* A. L. GIHON, M.D., *Medical-Director, United States Navy.*

[Read before the Naval Medical Society, Washington, D. C., December 2nd, 1886.]

The efficacy of the Mistura ferri et ammonii acetatis, (U. S. P.) in the treatment of a case of albuminuria incident to chronic Bright's disease, recently under my care, was so pronounced that I feel that it ought to be brought to the notice of the members of the Society.

The patient, an officer, was admitted into the United States Naval Hospital at Washington, D. C., 28th of March, 1886, having been invalided from the U. S. S. Nipsic at Montevideo, February 16th, as with "Febris remittens." He had been transferred from the U. S. S. Enterprise on the 9th of January, his disease being stated to be the result of exposure to malarial influences during a former long cruise on the China Station. During his stay at Montevideo, he was found to have albuminous urine, œdema of the lower extremities and dyspnœa.

At the Naval Hospital, New York, where he was first received on reaching the United States, his urine was noted as "smoky, variable in quantity, containing from one-sixth to one-twelfth of its volume of albumen, with granular and hyaline casts. There was general œdema with consequent dyspnœa; spasmodic breathing, interference with heart's action and pains in the extremities." He improved sufficiently to permit his transfer to Washington, at his own request, that he might be with his family, but the fatigue of travel proved more than he was able to undergo, and he arrived in a state of extreme prostration, his heart acting feebly and tumultuously, beating one hundred and forty to one hundred and sixty per minute—pulse scarcely discoverable at the wrist—temperature ninety-nine, features pinched, eyes pearly, skin dull yellow, surface cold, clammy sweat, tongue broad, moist, pale and cold to the touch, great præcordial distress, dyspnœa, excessive restlessness, moaning, hiccough, distention over lower pleuræ, tumid abdomen, almost complete suppression of urine, and incessant violent retching and vomiting.

He was put into a hot bath, dry cups applied over the loins, and the abdomen enveloped in hot turpentine stupes. Later the Mistura ferri et ammonii acetatis, the use of which had been commenced at the Naval Hospital at New York was administered in small doses alternately with boiled milk and lime water and repeated as often as it was vomited. This treatment, catharsis being at the same time established, was persisted in with such modifications as circumstances required. The irritability of the stomach gradually abated on the restoration of the secretion of urine, which on the 26th had increased in quantity to 300 c. c. during twenty-four hours, having specific gravity 1018 and containing ten per centum of albumen and on the 27th, to 500 c. c. specific gravity 1005, albumen six per centum; granular casts and fatty matter were abundant.

As the stomach and kidneys recovered their functions, the Basham's mixture was continued in larger doses at longer intervals, and on the 22d of May the patient was able to accompany his mother and sister to Missouri, arriving at their home on the 24th, after a rest at St. Louis. On the 25th he went under the professional care of Dr. Edward N. Small, of Sedalia, who wrote that he had accomplished the trip without much fatigue, complaining principally of dyspnœa. His urine amounted to about 800 c. c. in twenty-four hours, of specific gravity, 1018, containing "a considerable quantity of albumen and fatty casts." On July 24th Dr. Small writes: "His urine has specific gravity 1022, slightly acid, average quantity daily thirty-four ounces (nearly 1,100 c. c.), no casts and careful examination failed to detect the slightest trace of albumen. Improvement has been rapid during the last few weeks, his flesh has become firmer and of rosier color, all puffiness has disappeared from the face and swelling from the lower extremities. Some abdominal distention remains, but is much less than a week ago. The treatment by turpentine stupes, milk diet, with an occasional hydragogue cathartic and Basham's mixture has been adhered to. I am exceedingly glad to report so favorably."

This case, as also one other under my observation in a patient, whose antecedent history had a record, as this one, of alcoholic indulgence, demonstrates the usefulness of this very elegant combination of iron and ammonium acetate, the use of which, begun under unpromising conditions, was fortunately perservered in by the successive medical attendants with such satisfactory results.

